# Nano-Infrared Imaging of Primary Neurons

**DOI:** 10.3390/cells10102559

**Published:** 2021-09-27

**Authors:** Raul O. Freitas, Adrian Cernescu, Anders Engdahl, Agnes Paulus, João E. Levandoski, Isak Martinsson, Elke Hebisch, Christophe Sandt, Gunnar Keppler Gouras, Christelle N. Prinz, Tomas Deierborg, Ferenc Borondics, Oxana Klementieva

**Affiliations:** 1 Brazilian Synchrotron Light Laboratory (LNLS), Brazilian Center for Research in Energy and Materials (CNPEM), Campinas 13083-970, Sao Paulo, Brazil; joao.levandoski@lnls.br; 2 Attocube Systems AG, Eglfinger Weg 2, 85540 Munich, Germany; Adrian.Cernescu@neaspec.com; 3 Medical Microspectroscopy, Department of Experimental Medical Science, Lund University, 22180 Lund, Sweden; anders.engdahl@med.lu.se (A.E.); agnes.paulus@med.lu.se (A.P.); 4 Experimental Neuroinflammation Laboratory, Department of Experimental Medical Science, Lund University, 22180 Lund, Sweden; tomas.deierborg@med.lu.se; 5 Experimental Dementia Research, Department of Experimental Medical Science, Lund University, 22180 Lund, Sweden; isak.martinsson@med.lu.se (I.M.); gunnar.gouras@med.lu.se (G.K.G.); 6 Division of Solid State Physics and NanoLund, Lund University, 22100 Lund, Sweden; elke.hebisch@ftf.lth.se (E.H.); christelle.prinz@ftf.lth.se (C.N.P.); 7 Synchrotron SOLEIL, L’Orme des Merisiers, CEDEX, 91192 Gif Sur Yvette, France; christophe.sandt@synchrotron-soleil.fr (C.S.); ferenc.borondics@synchrotron-soleil.fr (F.B.)

**Keywords:** O-PTIR, s-SNOM, neuron, amyloid-beta, Alzheimer’s disease

## Abstract

Alzheimer’s disease (AD) accounts for about 70% of neurodegenerative diseases and is a cause of cognitive decline and death for one-third of seniors. AD is currently underdiagnosed, and it cannot be effectively prevented. Aggregation of amyloid-β (Aβ) proteins has been linked to the development of AD, and it has been established that, under pathological conditions, Aβ proteins undergo structural changes to form β-sheet structures that are considered neurotoxic. Numerous intensive in vitro studies have provided detailed information about amyloid polymorphs; however, little is known on how amyloid β-sheet-enriched aggregates can cause neurotoxicity in relevant settings. We used scattering-type scanning near-field optical microscopy (s-SNOM) to study amyloid structures at the nanoscale, in individual neurons. Specifically, we show that in well-validated systems, s-SNOM can detect amyloid β-sheet structures with nanometer spatial resolution in individual neurons. This is a proof-of-concept study to demonstrate that s-SNOM can be used to detect Aβ-sheet structures on cell surfaces at the nanoscale. Furthermore, this study is intended to raise neurobiologists’ awareness of the potential of s-SNOM as a tool for analyzing amyloid β-sheet structures at the nanoscale in neurons without the need for immunolabeling.

## 1. Introduction

Although Alzheimer’s disease is often regarded as part of natural aging, in fact, it is a progressive pathology in which a patient’s cognition worsens over time [1]. The “amyloid hypothesis” states that, based on observations, AD patients have amyloid plaques in their brains. Amyloid plaques are the buildup of β-sheet folded amyloid-β (Aβ) proteins that cause neurotoxicity. Therefore, eliminating Aβ from the brain should slow the onset of AD [2]. However, drugs targeting Aβ have not been effective, thus indicating that the molecular mechanisms of Aβ neurotoxicity are not entirely understood [3]. Aβ is processed from an amyloid precursor protein (APP) that is physiologically expressed in human nervous tissue; therefore, certain amounts of Aβ are always physiologically present. Under pathological conditions, Aβ undergoes structural changes and can form amyloid aggregates [4]. Immunofluorescence and immunoelectron microscopy are widely used to produce detailed images of amyloid proteins in cells or organs [5,6]. However, immunolabeling-based techniques are not always suitable for structural analysis since antibody generation requires prior knowledge of the targeted epitope structure; therefore, few structure-sensitive antibodies are available, and this limits the ability of immunolabeling to discriminate between different amyloid polymorphs that may be present in neurons [7,8,9].

Fourier-transform infrared microspectroscopy (μ-FTIR) is a conformation-sensitive method that can detect β-sheet structures by measuring the infrared (IR) absorbance of amyloid structures in tissues at the micron scale [10,11]. Importantly, β-sheet structures have specific IR spectral fingerprints and thus can be detected without the need for immunolabeling [12,13]. However, according to the Rayleigh criterion, within the range of 1000–2000 cm^−1^, the range needed to study amyloid structures, the spatial resolution is about 5−10 μm, which is not high enough to resolve amyloid structures at the subcellular level. The use of attenuated total reflection (ATR) IR microscopy can improve the spatial resolution to about 1 µm [14]; however, the sample must be in contact with the ATR crystal, which makes the use of this application challenging for adherent neurons.

Optical photothermal infrared (O-PTIR) [15] microspectroscopy overcomes the limitations mentioned above and does not require samples to be deposited or transferred onto the ATR crystal, and it has a high spatial resolution (sub-micron) and enhanced sensitivity [16,17]. O-PTIR is a structure-sensitive method that measures the IR photothermal response of a sample illuminated by a pulsed IR beam. The photothermal response is detected as a change in the sample reflectivity using a visible probe beam (532 nm laser), whose scattering is sensitive to the sample’s thermal expansion driven by the IR pulses, thus improving the spatial resolution (~500 nm) [15]. However, spatial resolution is still an obstacle to investigating amyloid aggregates due to their nanoscale size, typically 20–50 nm [18]. Therefore, nanoscale IR techniques such as scattering-type scanning near-field optical microscopy (s-SNOM) [19,20] can be a versatile tool to assess amyloid chemistry and morphology. s-SNOM is a surface-sensitive technique that is not limited by diffraction, as in methods such as μ-FTIR and O-PTIR. s-SNOM combines atomic force microscopy (AFM) and IR spectral imaging, where the AFM tip functions as a local probe to detect morphology and the scattering of optical fields [21,22].

Nanoscale mapping is achieved by recording the amplitude and phase of the IR light scattered by the near-field antenna (AFM tip) that scans a sample. While the sample is illuminated by broadband IR radiation, Fourier-transform spectroscopic measurement of the local scattered light can yield full IR spectra at a nanoscale spatial resolution [23,24,25,26,27]. Thus, s-SNOM provides a 20–30 nm spatial resolution, allowing for in situ structural identification and mapping of molecular structures.

In this study, we exploited s-SNOM to locate β-sheet structures directly in individual primary neurons from a validated transgenic mouse model of AD, APP/PS1 [28]. These AD transgenic mice express human amyloid precursor protein (APP) with the Swedish mutation and presenilin1 with L166P mutation, therefore cultured neurons produce human Aβ [28], and elevated β-sheet structural content can be found when these neurons are cultured for about 19 days [29]. Thus, using cultured primary APP/PS1 neurons as a well-characterized system, we validated s-SNOM as a helpful tool that can be used to study amyloid structures on the neuronal surface and in subcellular organelles. Specifically, we focused on label-free spectral imaging of β-sheet content in primary neurons cultured on 1 × 1 cm Si chips coated with 100 nm gold. Our proof-of-concept study shows that s-SNOM can be used to locate β-sheet structures associated with neuronal membranes at a nanometer resolution. Moreover, using the band intensities that correspond to lipids and proteins, we tested the chemical imaging of individual exosomes isolated from neurons from transgenic models of AD, which, to date, has not been reported. Thus, we present an attractive nanospectroscopic approach that can be used to study protein aggregation at the nanoscale.

## 2. Materials and Methods

### 2.1. Synchrotron-Based FTIR

Synchrotron-based μ-FTIR was performed at SOLEIL synchrotron facility (L’Orme des Merisiers, France) at the SMIS experimental station. Primary neurons grown for 19 days on 1 mm thick CaF_2_ (Eksama Optics, Vilnius, Lithuania) were imaged at room temperature using a Thermo Fisher Scientific Continuum XL FTIR equipped with a 32× magnification, 0.65 NA Schwarzschild objective. FTIR spectra were collected in transmission mode (1000−4000 cm^−1^). The spectral resolution was 4 cm^−1^, aperture dimensions were 6 × 6 µm, and 512 co-added scans were used. Background spectra were collected from the same window in a clean area closest to each measurement position. All measurements were performed at room temperature.

### 2.2. Optical Photothermal Infrared Measurements

For O-PTIR measurements, primary neurons were grown for 19 days on 1 mm thick CaF_2_ (Eksma Optics, Vilnius, Lithuania). We used a commercial mIRage^TM^ microscope (Photothermal Spectroscopy Corp., Santa Barbara, CA, USA) with a pulsed IR source and a tunable four-stage QCL device, scanning from 800 to 1800 cm^−1^ at an 80 kHz repetition rate. The probe was a CW 532 nm visible variable power laser; the photothermal effect was detected through the modulation of the probe laser intensity induced by the pulsed IR laser. Details about the fundamentals of the technique and the instrument itself can be found in the literature [17,30,31]. To generate data with a sufficient signal-to-noise ratio, spectra were averaged from at least 10 scans with an acquisition time of 1 sec per spectra. Background spectra were collected on an aluminized mylar reference sample. The collection parameters were: the spectral range of 1800−808 cm^−1^, reflection mode at 2 cm^−1^ spectral resolution, and a 500 nm step size. IR power set at 100% with~1.8 kW/cm^2^ average power density, calculated using the objective nominal spot size specified by the manufacturer as 450 nm and shortest available wavelength. To avoid photodamage, the probe power reaching the sample was set to ~2 mW. To remove laser chip transition effects and intensity jumps arising around the transition wavelength between the different QCL laser stages, spectra were “deglitched” using the PTIR Studio software. O-PTIR spectra were normalized and averaged; second-order derivation of the spectra was used to increase the number of discriminative features. The Savitzky−Golay algorithm with a 10-point filter and 3rd-order polynomial was employed in this process.

### 2.3. s-SNOM Nanoimaging and Nano-FTIR Measurements

For s-SNOM measurements, primary neurons were grown on AFM chips (Platypus Technologies, Fitchburg, WI, USA) for 12 or 19 days. s-SNOM measurements were carried out using a commercial s-SNOM nanoscope (NeaSNOM, Neaspec Attocube systems, Haar, Munich, Germany), operated in a soft tapping mode optimized for scanning biological samples. For nano-FTIR measurements, an AFM tip with an apex radius of 25 nm (NCPt-Arrow and PtSi-NCH, Nanoworld AG) was illuminated using the emission of an IR broadband laser source (Toptica, Graefelfing, Munich, Germany) and broadband synchrotron from the IR1 beamline at LNLS [24]. The light backscattered from the oscillating tip was analyzed with an asymmetric Michelson interferometer and detected with a mercury cadmium telluride detector [25,26,27]. Background spectra were collected from a clean area of the same sample and used for normalization to remove the instrumental response, similar to the classical FTIR technique. We used 12.5 cm^−1^ spectral resolution for all the spectra presented, with a 2 s/spectrum measurement time for the hyperspectral data set.

s-SNOM nanoimaging was performed using an interferometric detection scheme and a tunable QCL laser (Mircat, Daylight Solutions, Wausau, WI, USA) while collecting the AFM topography and stiffness information. The pseudoheterodyne detection principle allows the simultaneous recording of the optical phase and amplitude as a function of tip position on the sample, thus obtaining absorption and reflectivity maps at the wavelengths of interest emitted by the QCL laser superimposed with the topography map [21,32,33]. The average time for an image recording was 7 min at a spatial resolution of ~25 nm.

For the synchrotron-based s-SNOM, the spectral irradiance at the tip-sample stage was ~1 W/cm^−1^/cm^2^ in the mid-IR range. To avoid variability due to the tip, we used the same tapping mode AFM Probes (NanoWorld AG, Neuchâtel, Switzerland); to avoid AFM tip contamination, we regularly changed tips and recorded topography maps from a clean substrate after each sample.

### 2.4. Spectral Data Analysis

Orange software (University of Ljubljana, Ljubljana, Slovenia), OPUS (Bruker, Ettlingen, Germany), PTIR studio (Blue Scientific, Cambridge, UK), and NeaPlot software (Neaspec GmbH, Munich, Germany) were used to analyze FTIR, O-PTIR, and s-SNOM correspondingly. For µ-FTIR spectra, a linear baseline correction was applied from 1500 to 2000 cm^−1^. Spectra were averaged from at least 10 measurements and were normalized; second-order derivation of the spectra was achieved using the Savitzky−Golay algorithm with a 10-point filter and a polynomial order of three. The β-aggregation level of proteins was studied by calculating the peak intensity ratio between 1620 and 1640 cm^−1^, corresponding to β-sheet structures, and the maximum at 1656 cm^−1^, corresponding to α-helical content. Analysis of β-sheet load was conducted qualitatively by comparing values (1630/1650) from transgenic wild-type neurons using a t-test for comparing two groups; statistics with a value of *p* < 0.01 are considered significant. An increase in the 1620–1640 cm^−1^ component is considered a signature of amyloid fibrils [13].

### 2.5. Topographic Data Analysis

AFM height images were processed with the Gwyddion 2.53 software [34] by applying a flattening algorithm to remove the background slopes.

### 2.6. Primary Neuronal Cultures

Primary neuronal cultures were established following the ethical guidelines and approved by the Lund University Ethical committee (M46-16). Primary neurons were isolated from wild-type (#JAX 000664C57Bl/6J, Jackson Labs, Bar Harbor, ME, USA) APP/PS1 and APP-KO (#JAX 004133, Jackson Labs, Bar Harbor, ME, USA) mouse embryos on embryonic day 16, as described in reference [35], and seeded on 100 nm gold-coated Si chips. Before plating, gold-coated Si chips were coated with poly-d-lysine with molecular weight >300,000 (Sigma-Aldrich, Sweden) and then rinsed in autoclaved distilled water. Cell suspensions were plated in Dulbecco’s modified Eagle medium (DMEM) (Thermo Fisher Scientific, Gothenburg, Sweden) containing 10% fetal bovine serum (FBS) (Gibco, Thermo Fisher Scientific, Gothenburg, Sweden) and 1% penicillin–streptomycin; after 3–5 h, media were exchanged for FBS-free complete neurobasal medium. Primary neuronal cultures were maintained in a neurobasal medium supplemented with glutamine, B27, and penicillin–streptomycin (Thermo Fisher Scientific, Gothenburg, Sweden) after 19 days of culturing. To avoid artificial structural changes, neurons were fixed with 4% paraformaldehyde in phosphate buffer saline for 15 min, washed, and air-dried [36]. No differences in cell density or viability were observed between APP/PS1, WT, and APP-KO neurons. All the experiments were repeated 3–4 times; one embryo corresponded to one set of cultures. Neurons were grown directly on 100 nm gold-coated AFM chips (Platypus Technologies, Madison, WI, USA) and CaF_2_ windows (Eksma Optics, Vilnius, Lithuania).

Aβ treatment: Neurons were grown in 24-well plates for 19 days; before the Aβ treatment experiment, the old medium was removed, neurons were rinsed with in phosphate-buffered saline (PBS) (Thermo Fisher Scientific, 10010-015, Gothenburg, Sweden)., and 1 mL of fresh medium was added to each well. For the treatment, 4 µL of 250 µmol Aβ(1–42) was added to the well. Thus, the final concentration of Aβ(1–42) was 1 µmol. After 30 min incubation, neurons were rinsed with PBS and fixed with 4% PFA, washed, and kept in PBS at 4 °C until immunoreaction or washed with water and air-dried for spectroscopic experiments.

Trypsin treatment: Primary neurons were rinsed with PBS and incubated on ice for 10 min either with PBS alone or with 10 μg/mL trypsin (Thermo Fisher Scientific, Gothenburg, Sweden in PBS. The trypsin in the cultures was inactivated by rinsing neurons with 10% FBS and 1% penicillin–streptomycin in Dulbecco’s modified Eagle medium (DMEM, GE Healthcare Life Sciences, Uppsala, Sweden). The treated neurons were then washed with ice-cold PBS, fixed with 4% PFA, washed with water, and air-dried for μ-FTIR experiments.

### 2.7. Exosome Isolation and Analysis

Exosomes were purified from the cell culture medium by differential ultracentrifugation as described previously [37]. Briefly, neurons were cultured in an exosome-depleted medium. The collected medium was depleted of cells and cellular debris by low-speed centrifugation (10 min 5000× *g* at 4 °C). Exosomes were isolated by centrifugation of the collected supernatant at 100,000× *g* at 4 °C for 70 min. The resultant pellet was washed in PBS and centrifuged one more time for 70 min at 100,000×g at 4 °C. The quality of isolated material was checked by Western blotting with antibodies specific to the exosomal marker flotillin (Figure S1). Specifically, for quality analysis, after ultracentrifugation, pellets were re-suspended in phosphate buffer saline (PBS) with protease inhibitor and phosphatase inhibitor (Thermo Fisher Scientific, Gothenburg, Sweden) 20 µL per 10 cm Petri dish. For WB analysis, samples were mixed with 6% SDS loading buffer (*v/v*), heated at 95 °C for 5 min, and loaded into 10–20% tricine gels (Invitrogen, Gothenburg, Sweden). After electrophoresis, proteins were transferred to polyvinylidene difluoride membranes (Sigma-Aldrich, via Merck KGaA, Darmstadt, Germany). Membranes were boiled in PBS for 5 min, blocked in PBS containing 0.1% Tween-20 and 5% milk, and incubated with primary antibodies overnight and then with HRP-conjugated secondary antibodies for 1h. The immunoreaction was visualized by a chemiluminescence system (BioRad, Solna, Sweden).

### 2.8. Immunohistochemistry

PFA-fixed neurons were kept in a storage buffer composed of 30% sucrose and 30% ethylene glycol in PBS at −20 °C until use. Dual immunolabeling was performed as described in [29] using optimal working dilutions recommended by the manufacturer. Aβ 42 was visualized using monoclonal antibody 12F4 (#05-831-I, Merck, Stockholm, Sweden); amyloid fibrils were visualized with monoclonal antibody 82E1 (#10323, IBL America, Minneapolis, MN, USA). For post-synaptic labeling, rabbit polyclonal Drebrin antibody (#ab11068, Abcam, Cambridge, UK) and Map2 (#ab5392, Abcam, Cambridge, UK) were used. Secondary antibodies Alexa Fluor^®^ 405, 488, and 568 (Thermo Fisher Scientific, Gothenburg, Sweden) were used for confocal imaging. STARRED (Abberrior, Göttingen, Germany) was used as a secondary antibody for STED. For Aβ immunofluorescent labeling, we used APP-KO neurons that do not express Aβ as a negative control [35].

### 2.9. Confocal and STED Microscopy

Confocal images were obtained using a Leica TCS SP8 confocal microscope (Leica Microsystems) equipped with diode (405/405 nm) and argon (405, 488, 552, and 638 nm) lasers with an HP PL APO 63x/NA1.2 water immersion objective. Autoquant (MediaCybernetics, Rockville, MD, USA) was used for image deconvolution. STED images were obtained using an Abberior 2C STED 775 QUAD Scan system (Abberior Instruments GmbH, Goettingen, Germany). The STED signal was recorded using the 561 nm excitation laser, the 775 nm STED laser, and a (685/70) nm detection window, as described elsewhere [38].

### 2.10. Statistical Analysis

Statistical comparisons were made using a paired *t*-test for comparing two groups; as a variable, we used the ratio between 1656 cm^−1^, which corresponds to total protein, and 1630 cm^−1^, which corresponds to β-sheet structures. Statistics with a value of *p* < 0.01 are considered significant, and *** indicates a significant difference at *p* < 0.001. For the experiments, we used 3 embryos per genotype and 10–20 cells per condition. Data were assumed to fit a normal distribution without formal testing. Statistical analysis was performed using OriginPro 2020 (OriginLab Corporation, Northampton, MA, USA) software.

## 3. Results

**Figure 1:**
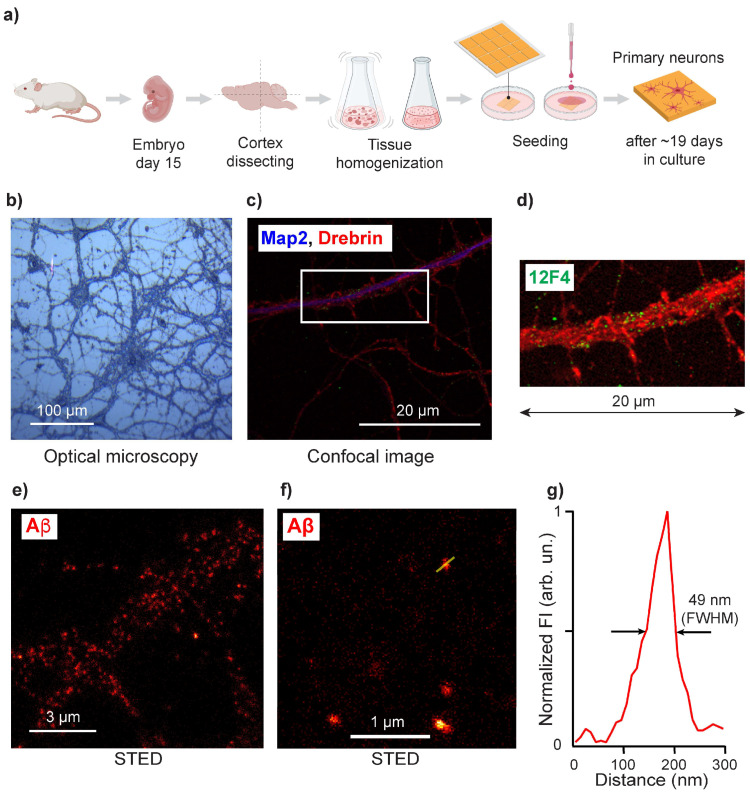
Sample preparation and characterization of intraneuronal Aβ aggregates. (**a**) Isolation of primary neurons. Cortex homogenate extracted from day 15 embryos was seeded on 100 nm gold-coated Si. After 19 days of growth and maturation in culture, neurons were fixed and air-dried. (**b**) Optical image of cultured APP/PS1 transgenic neurons on AFM substrate cultured for 19 days. A scanning electron micrograph of cultured neurons is shown in Figure S2. (**c**) Confocal micrograph of APP/PS1 transgenic neurons, where the blue channel corresponds to the neuronal marker Map2, and the red channel shows the post-synaptic marker Drebrin; Aβ42 labeled with 12F4 antibody is green. (**d**) Confocal micrograph of APP/PS1 transgenic neurons, where the green channel is used to map Aβ along the neurite labeled with Drebrin. (**e**,**f**) STED images of APP-KO neurons treated with Aβ(1-42), indicated by the red channel. (**g**) STED signal intensity profile of a single Aβ aggregate (shown in the middle panel) with a size of ca. 50 nm. Aβ size distribution is neurons is shown in Figure S3.

To detect β-sheet structures related to AD, we used cultured primary neurons carrying a human AD mutant APP gene. Neurons were seeded directly on the AFM chip and grown for 19 days in culture (Figure 1a,b). These neurons produce human Aβ(1–42) (Figure 1c,d), and with age, endogenous Aβ may aggregate and form β-sheet structures [29]. To avoid artificial influences on amyloid structures, neurons were fixed in 4% paraformaldehyde to crosslink two Aβ lysine ϵ-amino groups, thus preserving Aβ structures. Before measurements, neurons were air-dried [36]. Neurons were analyzed for the presence of Aβ(1–42) by immunofluorescence using the Aβ42-specific antibody 12F4 (Figure 1c,d). To estimate the size of Aβ aggregates, we used APP knock-out (KO) neurons that lack a functional amyloid precursor protein gene and are therefore free from Aβ. APP-KO neurons were treated with synthetic Aβ(1–42). To produce oligomeric Aβ, neurons were incubated with Aβ(1–42) at a final concentration of 1 µMol for 30 min, which is sufficient time for Aβ(1–42) to aggregate [29] and be partially absorbed by neurons [28].

Using STED, we observed that the sizes of immunofluorescent spots were around 50 nm (Figure 1e–g). Since APP-KO neurons lack Aβ, all the detected fluorescent signals used for STED quantification analysis were unambiguously attributed to exogenously added Aβ(1–42). Thus, because the average size of Aβ aggregates is 50 nm, nano-imaging approaches are necessary to investigate the structure of Aβ in neuronal cells.

**Figure 2:**
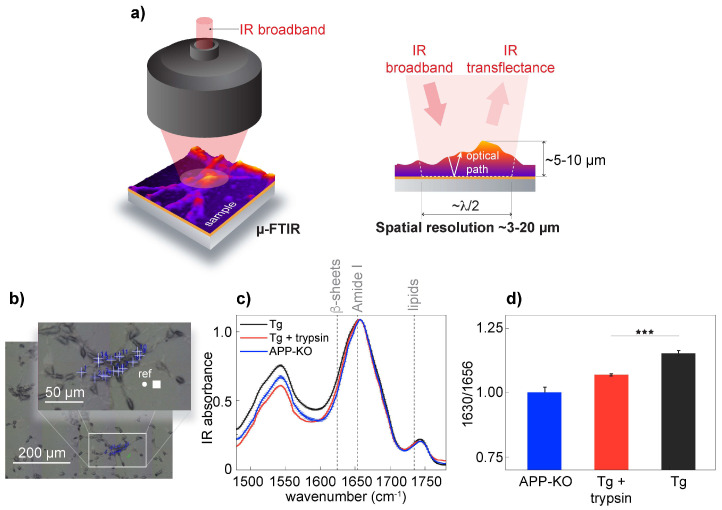
Fourier-transform infrared (µ-FTIR) point spectral analysis. (**a**) Schematic diagram of the working principle, penetration depth, and lateral resolution of µ-FTIR. For µ-FTIR reflectance geometry (right panel), the IR beam is focused on the sample, whose spot size is comparable to the illumination wavelength (diffraction-limited, 3–20 µm). The sampling depth is usually defined by the IR optical path inside the sample (~5–10 µm). (**b**) A bright-field visible-light image of cultured primary neurons. The white square in the digital insert shows the apertures used during the synchrotron-based µ-FTIR measurements. A white square indicates the aperture set to 8 × 8 μm^2^ and indicates the background position; Crosses show the position for spectra acquisition. (**c**) Averaged and normalized IR absorption spectra from untreated APP/PS1 transgenic neurons (red), APP/PS1 transgenic neurons (green), and APP-KO (black). Each point spectrum is an average of 256 spectral scans. FTIR spectra were acquired from different neurons (10 to 15 cells per condition), *** corresponds to *p* < 0.001 (**d**) Averaged and normalized second derivatives of the absorbance spectra mentioned in (**c**) show changes in the intensity at the 1630 cm^−1^ position (arrow). μ-FTIR spectra were acquired at 2 cm^−1^ spectral resolution with 256 averages in transmission mode. Statistics: a paired *t*-test for comparing two groups, *** indicates a significant difference at *p* < 0.001.

To validate s-SNOM, we confirmed the presence of aggregated Aβ in APP/PS1 transgenic neurons on the neuronal surface. It has been shown that Aβ can associate with the cell surface [39]. In our experimental setup, we investigated the presence of β-sheet structures in APP/PS1 transgenic, wild-type neurons and APP-KO neurons. For spectroscopic measurements, we used μ-FTIR and acquired spectra using an 8 × 8 µm^2^ aperture with a spatial resolution of approximately 3 μm in the Amide I frequency range (Figure 2a,b). Early empirical frequency-structure studies have found that β-sheets have an absorption band between 1625 and 1640 cm^−1^ [12,13]. Spectral analysis confirmed the presence of elevated levels of β-sheet structures in APP/PS1 transgenic neurons when compared to APP-KO neurons (Figure 2c,d); when trypsin digestion was used to remove surface-associated proteins, including amyloids [40], μ-FTIR data analysis showed a significant depletion of the β-sheet structural content as a drop of IR absorbance at 1630 cm^−1^, corresponding to β-sheet structures [13]. Previously, using FTIR, we demonstrated that wild-type and APP knock-out neurons have similar IR intensities at the bans corresponding to β-sheet structures, concluding that absorbance elevation at 1630 cm^−1^ can be assigned to aggregation-prone Aβ [16]. Since the intensities at 1630 cm^−1^ were similar between trypsin-treated APP/PS1 transgenic and APP-KO neurons, we conclude that trypsin removed from the cell surface proteins, including a vast amount of aggregated Aβ, thus confirming that aggregated Aβ was partially associated with the neuronal membrane of APP/PS1 transgenic neurons.

**Figure 3:**
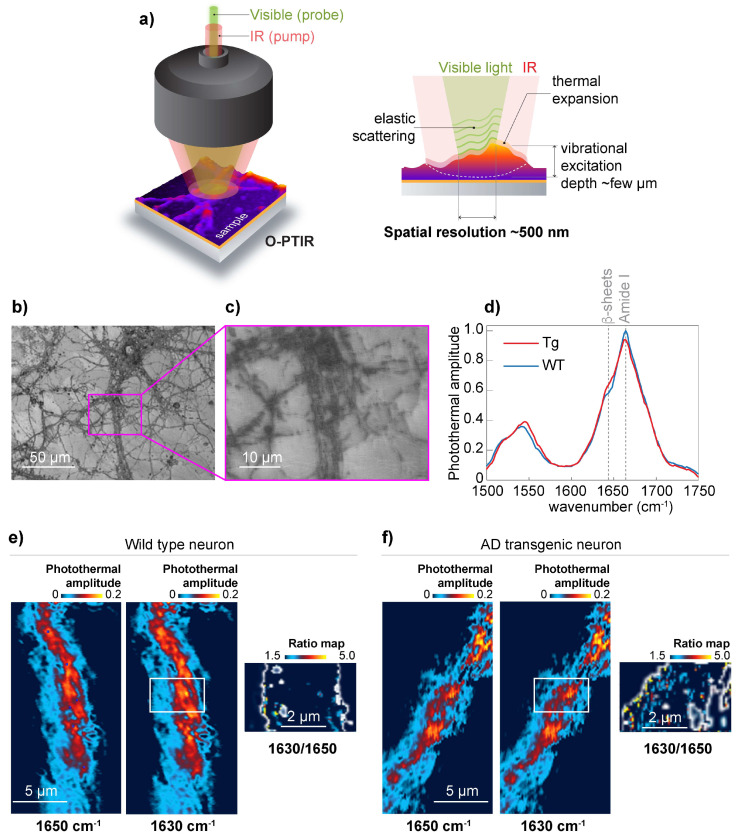
Optical photothermal infrared (O-PTIR) imaging of primary neurons. (**a**) Schematic diagram of the working principle, penetration depth, and lateral resolution for O-PTIR. The O-PTIR instrument employs two co-propagating beams: a 532 nm visible probe beam (shown as green in the illustration) and a tunable IR pump beam (shown as light red in the cartoon). The photothermal response is detected as the partial intensity loss of green light in response to the absorption of a pulsed IR beam. Thus, the spatial resolution is enhanced to ~500 nm. In O-PTIR (right panel), visible light is used as a probe; visible light scattering evolution is connected to the sample’s volume expansion produced by IR illumination (pump). The visible light scattering is then translated to the spectral response, enabling an IR probe, whose lateral resolution, ~500 nm, is defined by the visible probe (in this case, green light). Although the elastic scattering is limited to the surface, the penetration depth of the O-PTIR is defined by the reach of the IR beam (a few microns). (**b**) Representative optical image of cultured primary neurons grown on the CaF_2_ using 10× objective. (**c**) Zoom-in of the area indicated in (b) using 40× objective. Dots indicate representative O-PTIR spectra locations. (**d**) Averaged and normalized IR spectra were recorded from wild-type neurons (blue) and APP/PS1 transgenic neurons (red). Spectra were acquired at 2 cm^−1^ spectral resolution with 50 averages in reflection mode. (**e,f**) O-PTIR maps were acquired at frequencies 1650 cm^−1^ and 1630 cm^−1^ from wild-type and APP/PS1 transgenic neurons, respectively. Ratio maps are used to locate β-sheet structures (1630 cm^−1^) along the neurons. Mask was rendered using threshold set to 0.03 a.u. of photothermal amplitude using 1650 cm^−1^ map; white line separates threshold. (Figure S4).

To achieve 500 nm spatial resolution in the chemical mapping of the distribution of β-sheet structures, we used O-PTIR [16] schematically shown in Figure 3a. For O-PTIR experiments, we used wild type and APP/PS1 primary neurons grown on CaF_2_. The neurons were cultured for 19 days, and neurons with a high degree of neuronal process branching were selected for the study. (Figure 3b,c) Acquired O-PTIR spectra show an increase in the photothermal amplitude at 1630 cm^−1^, which is attributed to β-sheet structures [12] and considered to be the result of elevated β-sheet structural content in APP/PS1 transgenic neurons (Figure 3d). Next, we recorded O-PTIR maps at 1650 cm^−1^ and 1630 cm^−1^ from wild-type neurons (Figure 3e) and APP/PS1 transgenic neurons (Figure 3f). To normalize the results to the amount of protein, we acquired O-PTIR images at 1650 cm^−1^ and calculated the ratio map (1630 cm^−1^/1650 cm^−1^). The ratio map in Figure 3f shows the elevation of β-sheet structural content compared to the corresponding ratio map calculated for wild-type neurons. Importantly, the ratio map calculation was possible due to the remarkable baseline stability of the O-PTIR spectra, which are mainly impervious to the scattering [15] that characterizes μ-FTIR. For example, in conventional µ-FTIR transmission measurements, acquiring information at many wavelengths would be necessary to correct baseline drifts caused by IR scattering. Thus, using O-PTIR, we confirmed the presence of β-sheet structures distributed along the neurites of APP/PS1 transgenic neurons.

**Figure 4:**
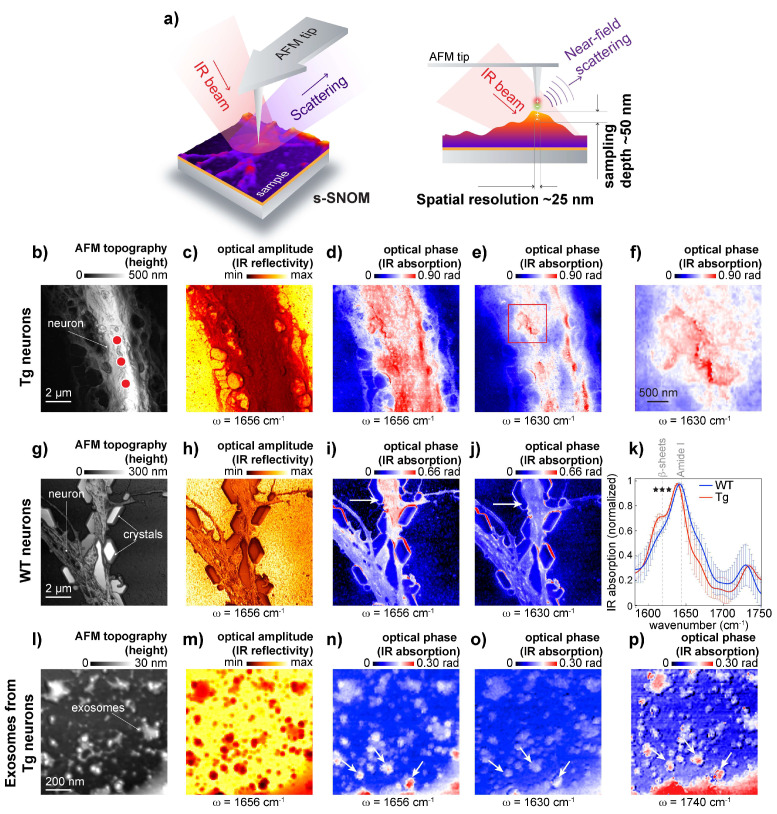
Scattering-type scanning near-field optical microscopy (s-SNOM) nanoimaging and nanospectroscopy. (**a**) Schematic diagram of the working principle, penetration depth, and lateral resolution for s-SNOM. In s-SNOM, a metallic AFM tip acts as an antenna for the incident IR radiation, whose scattering carries local information (or dielectric response) of the material’s surface (left panel). The tip-confined fields in s-SNOM are strongly attached to the metallic tip (evanescent fields); therefore, both lateral resolution and sampling depth are comparable to the tip radius: in this case, their values are ~25 nm and ~50 nm, respectively. (**b**–**f**) s-SNOM measurements of individual APP/PS1 transgenic neurons cultured on the AFM substrate: 10 × 10 µm^2^ AFM topography map of individual neurons with the dots indicating representative locations for spectra acquisition. The topography amps are followed by corresponding reflectivity and optical phase (IR absorption) images acquired at 1656 cm^−1^, 1630 cm^−1^, and digital zoom-in of the area indicated by the red square in 1630 cm^−1^ optical phase image to show amyloid patches on neuronal surface. (**g**–**j**) s-SNOM measurements of wild-type neurons cultured on the AFM substrate: 10 × 10 µm^2^ AFM topography map of individual neurons, followed by the corresponding reflectivity image and optical phase (IR absorption) images acquired at 1656 cm^−1^ and 1630 cm^−1^. (**k**) Averaged and normalized s-SNOM nano-FTIR broadband spectra recorded from wild-type (blue) and APP/PS1 transgenic neurons (red). Vertical dashed lines indicate spectral shoulders at around 1656 cm^−1^, 1630 cm^−1^, and 1740 cm^−1^. Each point spectrum is an average of 15–30 spectral scans. Error bars show standard deviation. s-SNOM spectra were acquired from different neurons (3 to 5 cells per genotype, using at least 3 independent preparations of neuronal cultures). Statistics: a paired t-test for comparing two groups; *** correspond to *p* < 0.001. (**l**–**p**) s-SNOM measurements of exosomes isolated from cultured APP/PS1 transgenic primary neurons: 1 × 1 µm^2^ AFM topography map of exosomes deposited on AFM substrate, followed by the corresponding reflectivity image and optical phase (IR absorption) images acquired at 1656 cm^−1^, 1630 cm^−1^, and 1740 cm^−1^. White arrows indicate lipid–protein-rich spots that were identified as exosomes.

To further study if β-sheet structures are associated with the neuronal membrane, we used s-SNOM, a technique based on AFM and IR nanoscopy [26,41]. In s-SNOM, the probing volume is defined by the AFM tip geometry [26], resulting in an approximate sampling depth of 50 nm (Figure 4a). This enabled the recording of high-resolution topography (Figure 4b,g,i), optical amplitude images that feature the local IR reflectivity coefficient of the neuron (Figure 4c,h,m), and optical phase images that correspond to IR absorbance maps recorded from APP/PS1 transgenic neurons representing IR absorbance at the frequencies 1650 cm^−1^ and 1630 cm^−1^ (Figure 4d,e), Figure 4f show patches of β-sheet structures on the neuronal surface.

To further study protein aggregates associated with the neuronal membrane, we used s-SNOM, a technique based on AFM and IR nanoscopy [21]. In s-SNOM, the AFM tip geometry defines the probing volume, resulting in an approximate sampling depth of 20–50 nm [26], as schematically shown in Figure 4a. This enabled the recording of high-resolution topography (Figure 4b,g,i), optical amplitude images that feature the local IR reflectivity coefficient of the neuron (Figure 4c,h,m), and optical phase images that correspond to IR absorbance maps representing IR absorbance at the frequencies 1650 cm^−1^ and 1630 cm^−1^. Maps that were recorded from APP/PS1 transgenic neurons show patches of β-sheet structures on the neuronal surface (Figure 4d–f) when compared with IR maps recorded from wild-type neurons (Figure 4i,j). Interestingly, a 1630 cm^−1^ signal can be observed on the edges of NaCl crystals that were intentionally left to demonstrate the importance of washing steps for the preparation of adhered cells for SNOM measurements. Here is worth mentioning that when microspectroscopy is used to study heterogeneous samples like neurons, intensity normalization is needed to consider sample thickness. However, since s-SNOM signal penetration depth is defined by AFM tip geometry, ratio map calculation becomes unnecessary. To further assess β-sheet structures associated with the neuronal membrane, we measured IR absorption on the neuronal membrane. The spectral analysis confirmed the presence of elevated levels of β-sheet structures in APP/PS1 transgenic neurons when compared to APP-KO neurons (Figure 4k). Thus, s-SNOM near-field phase images can show elevated content of β-sheet structures associated with the cellular membrane without mathematical processing.

It has been demonstrated that those exosomes, extracellular organelles, play a role in AD [42], and it has been hypothesized that exosomes can spread toxic Aβ and between cells. Because of vesicle size, 50–150 nm in diameter, we investigated whether s-SNOM can be used for the characterization of exosomes isolated from AD and wild-type neurons. To isolate exosomes, we used differential ultracentrifugation of conditioned media, which has been successfully used to isolate Aβ-positive vesicles from APP/PS1 transgenic neurons [37]. We evaluated the quality of the exosome isolation by Western blot using specific antibodies: exosome positive flotillin and exosome negative Lamp1 used to [28], as shown in Figure S1. For s-SNOM experiment, the exosomal precipitate was deposited on the AFM surface and air-dried. In our study, we identified exosomes as 50–150 nm structures with a positive contrast in the s-SNOM near-field phase images acquired at 1650 cm^−1^ (proteins) and 1740 cm^−1^ (lipids) (Figure 4l-p). Surprisingly, our data show the absence of elevated β-sheet structural content in the exosome preparation (Figure 4o). The absence of elevated β-sheet structural content could be explained by the increased fragility of AD exosomes; it can also indicate that the exosomes carried β-sheet free Aβ or spread of AD pathology goes via a non-exosomal pathway. Therefore, further study is required to understand whether exosomes can carry aggregated Aβ from diseased neurons to healthy cells, thus spreading the pathology. Another interesting observation is the presence of lipid-free protein clumps in the exosomal isolate. This indicates that the classical protocol used for the isolation of exosomes should be improved by including a purification step to remove these lipid-free protein aggregates, however, s-SMON can be used to differentiate between lipid enrich vesicles and protein clumps.

## 4. Discussion

AD is a very complex pathology, and to date, no consensus on the molecular mechanisms of amyloid neurotoxicity has been reached. It has been reported that intraneuronal Aβ accumulation can cause synaptic pathology and the enlargement of MVB [28,43]. It has been shown that different conformations of amyloid beta can induce neurotoxicity in neurons by distinct mechanisms [5]. However, the molecular mechanisms behind neuronal damage induced by Aβ aggregation in neurons are not well understood. In our previous work [44], we demonstrated that μ-FTIR and O-PTIR can be used to study molecular structures in primary neurons that produce aggregation-prone amyloid proteins such as Aβ. Here, we demonstrate that s-SNOM can simultaneously characterize the morphology and the complex optical (reflectivity and absorption) responses of the sample surface with a spatial resolution of ~50 nm. For example, in Figure 4g, phosphate saline crystals are observed in the AFM topography map of an individual neurite. These crystals demonstrate that a washing step with distilled water is needed for sample preparation to avoid the formation of salt crystals that can mask neurons and interfere with data analysis. In our demonstration study, NaCl crystals were used not to analyze SMOM data but left to show the difference between inorganic and organic substrates (Figure 4i,j). Such illustration may help SNOM data interpretation in biomedical experiments. Complementing AFM imaging, the s-SNOM optical amplitude and phase reveal the local IR reflectivity and absorption, respectively, of the neuron at 1656 cm^−1^ and 1630 cm^−1^.

Using s-SNOM, we detected β-sheet-enriched structures on the neuronal surface. Notably, the presence of β-sheet-enriched structures in AD cultured neurons was confirmed by μ-FTIR and O-PTIR, thus validating the results obtained by s-SNOM. Further, using s-SNOM, we examined protein structures in exosomes. It has been shown that exosomes isolated from APP/PS1 primary neurons contain Aβ [44]; however, the conformational states of Aβ cannot be assessed using WB or immunochemistry due to the chemical processing in these methods, which may alter Aβ conformations. Therefore, to address conformational states of Aβ, nanospectroscopic approaches are needed. We demonstrate that by using s-SNOM, we can select exosomes deposited on the surface (via a specific band that corresponds to lipids). Analyzing s-SNOM spectra, we did not detect β-sheet structures in these isolated exosomes, but because the presence of Aβ has been previously shown in exosomes [44], it could indicate that the exosomes carried β-sheet free Aβ. However, more experiments are needed to investigate why β-sheet-enriched structures were not detected in exosomes isolated from APP/PS1 transgenic neurons by a sequential centrifugation protocol and determine if modifications of the protocol are needed to preserve exosomes that may carry aggregated Aβ.

Further advances in imaging data processing are also necessary; therefore, in this study, we implemented one of the options for s-SNOM. In the s-SNOM measuring modality, we used a broadband synchrotron-based IR source instead of a tunable single-frequency QCL to illuminate the AFM tip. Then, the IR scattered from the tip location is Fourier transform of an interferogram into a broadband IR spectrum, allowing the collection of point spectra from a location matrix over the sample and a hyperspectral data set (Figure S5a). This HS data set comprises local IR reflectivity (Re[δ]) and IR absorption (Im[δ]) for a range of illumination frequencies (w) over an x-y point matrix (x, y, w, Re[δ], Im[δ]). It is worth noting that Im[δ] correlates directly with FTIR absorbance [41]. With this data set, it is possible to reconstruct the IR reflectivity or narrowband absorption images (Figure S5b–e), for example, to identify the exact peak position of β-sheets.

In summary, the main scientific achievement in this study is the successful application of s-SNOM to detect β-sheet structures in a single neuron. We demonstrate that s-SNOM can be used to image primary neurons in a straightforward manner, with no additional sample preparation being required. A high-resolution integrated bright-field microscope allows the detection of the areas of interest. The soft tapping operation mode of the s-SNOM microscope and the high sensitivity of the pseudo-heterodyne optical detection allow high-resolution topographic and optical measurements to be acquired at low laser power and with short measurement times. Most of the nano-FTIR spectra show characteristic absorption bands in less than 3 min of measurement time, demonstrating the high sensitivity of s-SNOM. Protein spectra show characteristic amide II bands with a spectral resolution of 3.3 cm^−1^. We did not detect surface damage in the investigated neurons (neurons were scanned several times, and we did not observe changes in spectra or topography). Recorded nano-FTIR spectra on a large population of neurons could indicate statistical differences in the amide band spectra of proteins, correlated with single-frequency absorption images at 1656 and 1630 cm^−1^, highlighting the distribution of α-helix and β-sheet structures, respectively. However, a systematic approach is required to explore the composition of a single amyloid oligomer in neurons. This approach should involve several important steps: a correlative approach with O-PTIR mapping and s-SNOM to assess a sufficient number of neurons, together with a collection of hyperspectral maps and PCA analysis. Simultaneous s-SNOM combined with O-PTIR could be applied to specifically detect amyloid oligomers and explore their structures, revealing new possibilities for detecting the neurotoxicity relationship. Finally, the further development of s-SNOM and O-PTIR for measurements in a liquid environment to study amyloid aggregation and propagation in living neurons suggests new possibilities for identifying the origin of AD.

## Data Availability

The data that support the findings of this study are available from the corresponding author upon reasonable request.

## Supplementary Materials

The following are available online at https://www.mdpi.com/article/10.3390/cells10102559/s1, Figure S1: Characterization of exosomal preparation, Figure S2: SEM sample characterization, Figure S3: Aβ-size distribution, Figure S4: Mask rendering, Figure S5: Example of s-SNOM hyperspectral nanoimaging.
