# Supplementary material for: Nano-Infrared Imaging of Primary Neurons
Source: Cells. 2021 Sep 27;10(10):2559. doi: 10.3390/cells10102559 (PMC8534030; doi:10.3390/cells10102559)
Supplement: Supplementary file 1 [file cells-10-02559-s001.zip › cells-1312906-supplementary.pdf]

Supplementary Information

Nano-infrared imaging of primary neurons

Raul O. Freitas [1,*], Adrian Cernescu [2], Anders Engdahl [3], Agnes Paulus [3,4], João E. Levandoski [1], Isak Martinsson [5], Elke Hebisch [6], Christophe Sandt [7], Gunnar Keppler Gouras [5], Christelle N. Prinz [6], Tomas Deierborg [4], Ferenc Borondics [7], Oxana Klementieva [3,*]

[1] Brazilian Synchrotron Light Laboratory (LNLS), Brazilian Center for Research in Energy and Materials (CNPEM), 13083-970 Campinas, Sao Paulo, Brazil; joao.levandoski@lnls.br (J.E.L.); raul.freitas@lnls.br (R.O.F.)
[2] Attocube Systems AG, Eglfinger Weg 2, 85540 Munich, Germany; Adrian.Cernescu@neaspec.com
[3] Medical Microspectroscopy, Department of Experimental Medical Science, Lund University, 22180 Lund, Sweden; anders.engdahl@med.lu.se (A.E.); agnes.paulus@med.lu.se (A.P.), oxana.klementieva@med.lu.se (O.K.)
[4] Experimental Neuroinflammation Laboratory, Department of Experimental Medical Science, Lund University, 22180 Lund, Sweden; tomas.deierborg@med.lu.se
[5] Experimental Dementia Research, Department of Experimental Medical Science, Lund University, 22180 Lund, Sweden; isak.martinsson@med.lu.se (I.M.); gunnar.gouras@med.lu.se (G.K.G.)
[6] Division of Solid State Physics and NanoLund, Lund University, Lund 22100, Sweden; elke.hebisch@ftf.lth.se (E.H.); christelle.prinz@ftf.lth.se (C.N.P.)
[7] Synchrotron SOLEIL, L'Orme des Merisiers, CEDEX, 91192 Gif Sur Yvette, France; christophe.sandt@synchrotron-soleil.fr (C.S.); ferenc.borondics@synchrotron-soleil.fr (F.B.)
* Correspondence: raul.freitas@lnls.br (R.O.F.); oxana.klementieva@med.lu.se (O.K.)

Keywords: O-PTIR; s-SNOM; neuron; Amyloid-beta; Alzheimer's disease

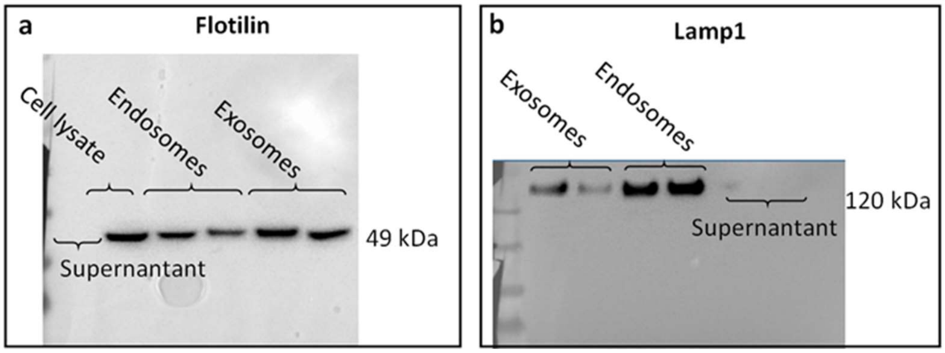

Figure ***S*1.** Characterization of exosomal preparation. Representative Western blots of cell lysate, supernatant, and fractions containing exosomes and endosomes are shown. (**a**) Representative Western blot with flotillin, a marker of exosomes. (**b**) Representative Western blot with Lamp1, a marker of endosomes.

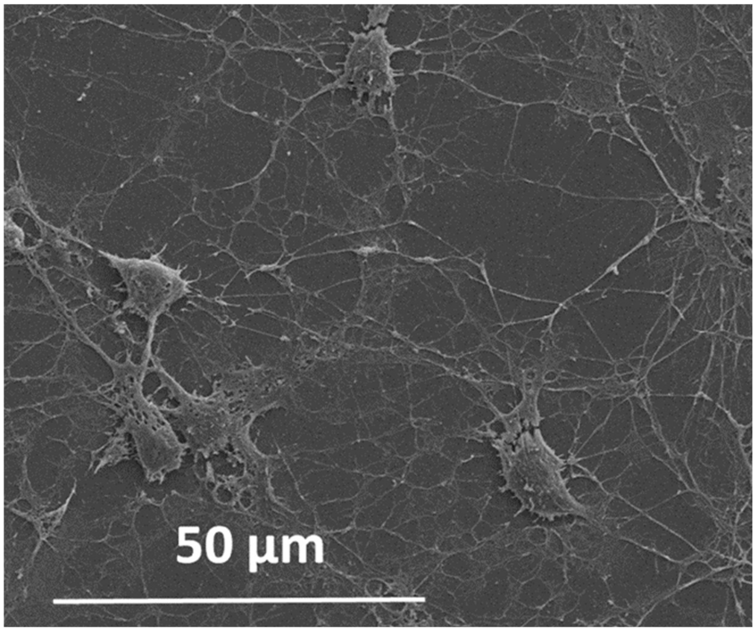

Figure S2. Sample characterization**.** Primary neurons were seeded on 100 nm gold-coated chips. After 19 days of growth and maturation, neurons were fixed with 4% paraformaldehyde in phosphate buffer saline for 15 min at room temperature, washed, and air-dried. Examining the neurons using surface scanning electron microscopy confirmed that air-drying did not affect the quality of the sample.

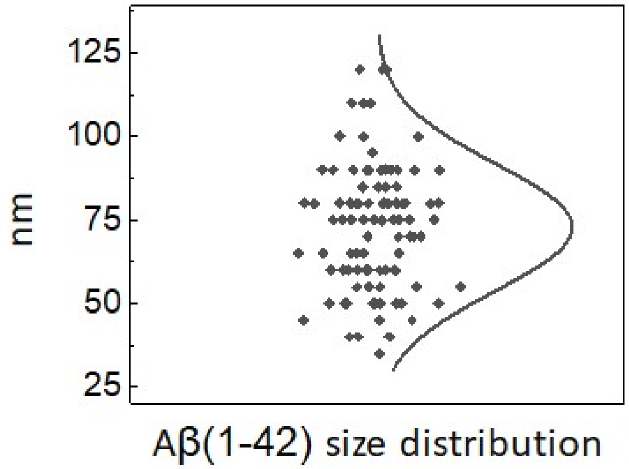

Figure S3. Aβ-size distribution. To calibrate STED micro-scope we used 20 nm fluorescent beads, Ø = 25 nm. The plot represents size distribution of Aβ(1–42) particles measured by S TED.

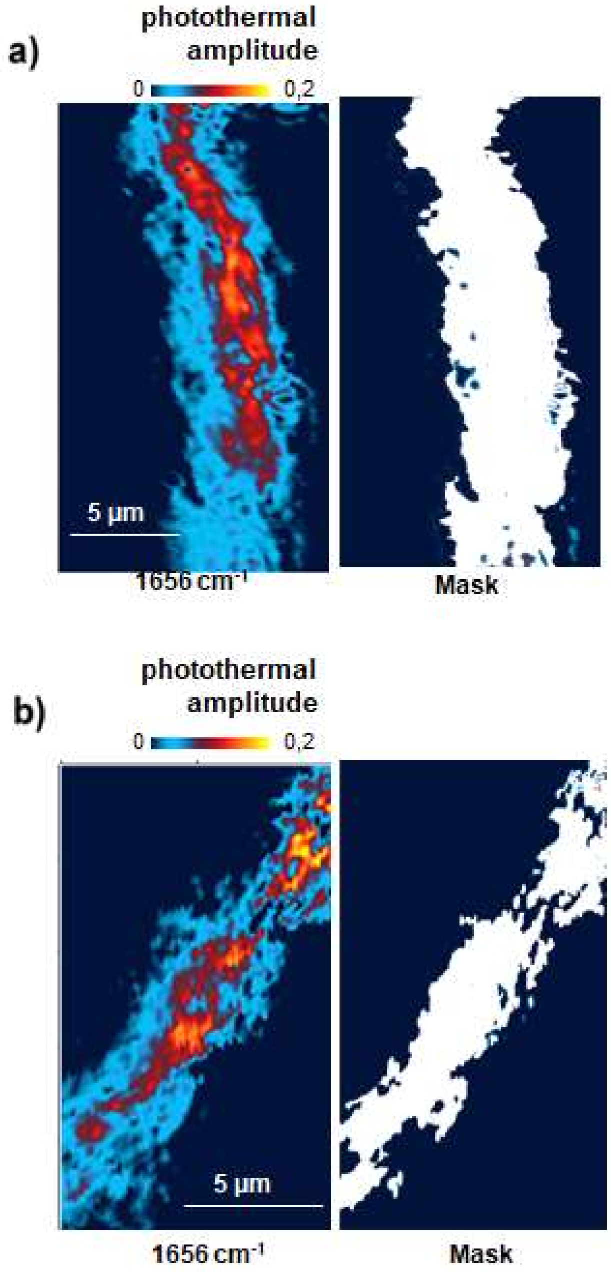

Figure S4. Mask rendering. O-PTIR maps were acquired from wild-type (**a**) and AD transgenic neurons (**b**). Blue-yellow gradient indicates elevations of the intensities at 1650 cm$^{-1.}$ Right panels: asks which were rendered using threshold set to 0.03 a.u. of photothermal amplitude of 1650 cm^{-1} map.

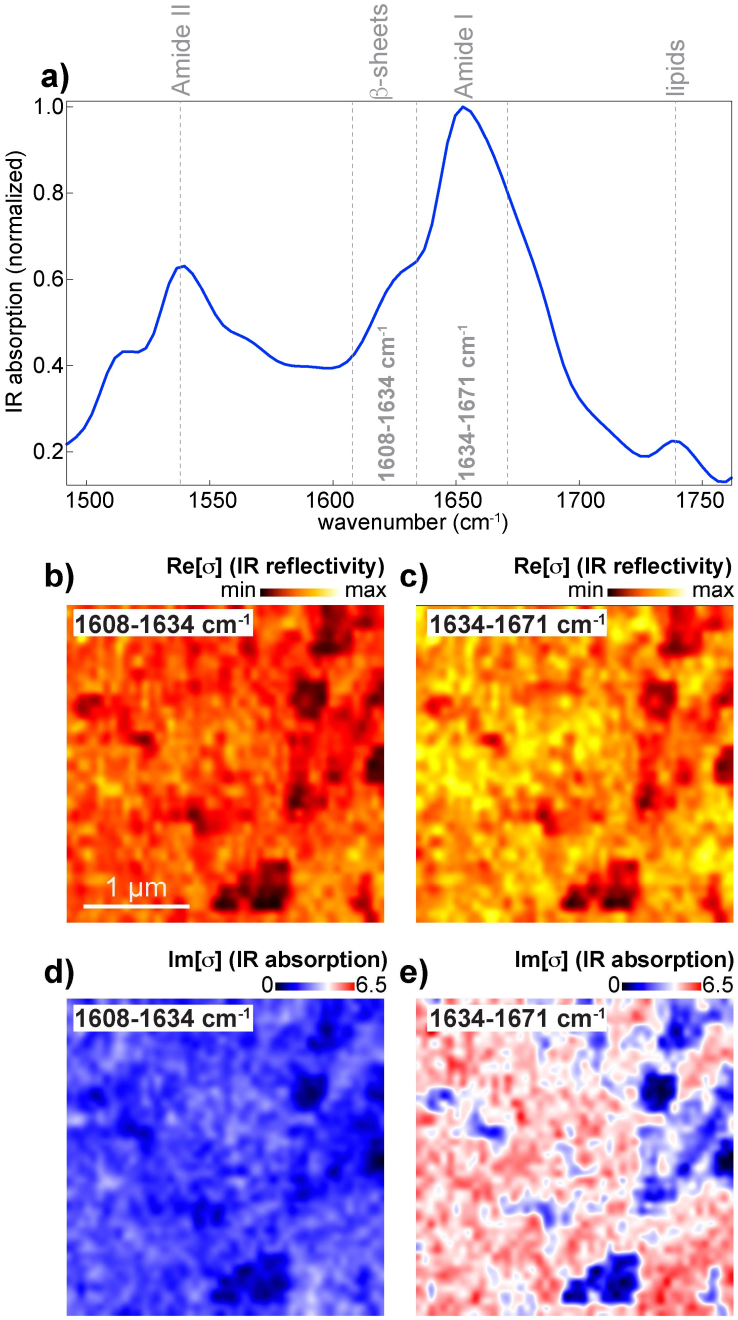

Figure S5. Example of s-SNOM hyperspectral nanoimaging. (**a**) Point spectrum from an arbitrary pixel of the HS map indicating the spectral ranges 1608–1634 cm^{-1} and 1634–1671 cm^{-1} used for nanoimaging analysis. (**b**–**c**) Nanoscale IR reflectivity integrated across the spectral ranges 1608-1634 cm^{-1} and 1634-1671 cm^{-1}, respectively, reconstructed from a 3 × 3 μm^2 HS map of wild-type neurons. (**d**–**e**) Nanoscale IR absorption images integrated within the spectral ranges 1608–1634 cm^{-1} and 1634–1671 cm^{-1}, respectively.
